# Editorial: Repurposed Drugs Targeting Cancer Signaling Pathways: Clinical Insights to Improve Oncologic Therapies

**DOI:** 10.3389/fonc.2021.713040

**Published:** 2021-06-23

**Authors:** Carlos Pérez-Plasencia, Teresita Padilla-Benavides, Eduardo López-Urrutia, Alma D. Campos-Parra

**Affiliations:** ^1^ Laboratorio de Genómica Funcional, Unidad de Biomedicina, Facultad de Estudios Superiores Iztacala, Universidad Nacional Autónoma de México, Tlalnepantla, Mexico; ^2^ Laboratorio de Genómica, Instituto Nacional de Cancerología (INCan), Mexico City, Mexico; ^3^ Department of Molecular Biology and Biochemistry, Wesleyan University, Middletown, CT, United States

**Keywords:** repurposed drugs, cancer, oncologic therapies, cancer research, clinical insights

The analyses of drug development from different standpoints show how resource-intensive this process is, with two main disadvantages: time and cost. It takes 11 to 14 years to develop a pharmaceutical product, with a financial cost currently estimated around 161-1,800 million dollars (1). Bypassing these limitations has made drug repositioning one of the most burgeoning areas in pharmacology over the past decade. Drug repositioning finds new applications of existing drugs by testing them against diseases unrelated to their initial use; so, the availability of complete data on pharmacology, formulation, safety, and adverse effects reduces development time and cost. However, it also has disadvantages: managing patents, intellectual property, investment, market demand, and even production technology (2). Regardless, drug repositioning poses a fascinating challenge with the potential to improve human health, in particular for the treatment of various types of cancer, but also a complex challenge in the legal and regulatory fields (3).

Drug repurposing has captivated the cancer research community due to the increasing demand for new anticancer drugs. Although there are several treatments, such as chemotherapy and targeted therapies, cancer is characterized by the eventual development of resistance or lack of response to these drugs and medications, making the design of new drugs against cancer a flourishing area of study. In this regard, drug repositioning is an attractive research area that has gained tremendous popularity. Oncology has taken advantage of existing, well-characterized, widely-used, non-cancer drugs and successfully tested them as anticancer agents (4–6). To get an overall picture, we searched the Medline (pubmed.ncbi.nlm.nih.gov) and ClinicalTrials.gov (clinicaltrials.gov) databases for reports indexed under “cancer” and “drug repositioning”. We found an evident increase the number of studies and clinical trials focused on drug repurposing in cancer since 2010 in both databases (**Figure 1** and **Supplementary File 1**) . Thus, we confirm that this strategy has yielded enriched oncology’s vision for treatment of cancer patients. Use of drugs approved for diabetes and hypertension, such as metformin and statins, for cancer clearly exemplifies this. Statins improved the survival in lymphoma patients (4). Metformin improved survival in type 2 diabetic patients with ovarian cancer (5). Also, beneficial anticancer effects or metformin has been shown in breast cancer patients (6). On the other hand, hydralazine, which is a typical antihypertensive drug, is employed in metastatic cervical and ovary cancer phase III clinical trial (NCT00532818 and NCT00533299, respectively). Likewise, is employed in phase II clinical trial that including breast cancer patients (NCT00395655) and in solid tumors to overcome chemotherapy resistance (NCT00404508). However, the dark side of non-oncological drugs, i.e., without single-agent activity in cancer, carries therapeutic failure risk. For instance, in preclinical studies of chloroquine, a drug originally intended to prevent or treat malarial infections, as anticancer drug have shown positive therapeutic effect; nevertheless, parameters, doses, animal models and tumor types differ strongly between studies, complicating the interpretation of the results and highlighting the need for further clinical investigations (7).

**Figure 1:**
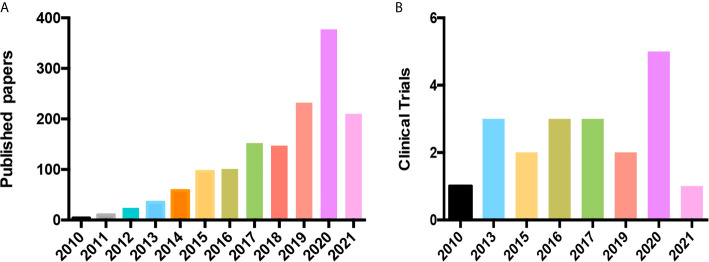
Drug repositioning in cancer has increased its relevance and popularity in recent years. **(A)** Papers published in PubMed in the last decade **(B)** Clinical Trials of the last decade.

Despite the efforts made, further research is still required to advance interventional approaches as well as to accelerate the introduction of drug repositioning in the clinic, improving treatments for cancer patients. The topic is vast, and the scientific community is focused on repositioning drugs to expand and improve cancer treatment. We appreciate the interest of the researchers to participate in this topic, in which ten manuscripts were collected (one original research and nine reviews). The research presented in this topic provide valuable information and insights on novel therapeutic options for cancer with existing drugs, facilitating their use in clinical practice. A short description of these manuscripts follows.

In their original research, Medina Jiménez and Monroy-Torres implemented an individualized nutritional intervention in cervical cancer patients treated simultaneously with radiotherapy. They reported the effect of personalized nutrition on the maintenance of muscle mass, weight, hemoglobin levels, and a decrease in gastrointestinal adverse effects, favoring the radiotherapy treatment outcome. Moreover, the authors suggested implementing an individualized nutritional intervention in cervical cancer patients treated with repurposing drugs to improve their efficacy and, therefore, the quality of life of oncology patients.

Martinez-Escobar et al., in their review, recommended the use of drug repurposing combined with CRISPR-dCas9-based artificial transcription factors (ATFs), as a viable alternative cancer treatment to reduce mortality. Strikingly, CRISPR-dCas9-based ATFs can manipulate DNA and modify target genes, activate tumor suppressor genes, silence oncogenes and tumor resistance mechanisms for targeted therapy. In cancer research, it is imperative to identify new drug combinations that generate synergistic effects and thereby achieve more efficient therapies.

Another strategy to treat cancer is nanomedicine, which is based on glycosylated nanoparticles (NPs). NPs can carry both cancer-targeting molecules and drugs and deliver them precisely, avoiding the severe side effects derived from nonspecific drug delivery in standard chemotherapy treatments. They bind to receptors overexpressed by tumor cells, such as lectin receptors, glucose transporters (GLUT), and glycosylated immune receptors of programmed cell death. In this regard, Torres-Pérez et al. reviewed crucial nanomedicine innovations to discover more specific cancer receptors and new glycan-based ligands or repurposed drugs against these receptors as potential opportunities for cancer therapy, prevention, pathological imaging, and theranostics.

Regarding drug resistance, Hu and Carraway discussed the role of cationic amphiphilic drugs such as antidepressants, antibiotics, antiarrhythmics, and diuretics to be repurposed to trigger lysosomal cell death (LCD) and lysosomal membrane permeabilization (LMP) within therapy-resistant tumor cell populations in their review.

Repositioning molecular strategies to fight cancer are also being studied. Montaño-Samaniego et al. reviewed gene therapy targeted toward cancer- and tumor-specific promoters. The authors focused on cancer suppressors and suicide genes by employing diverse experimental strategies, such as prevention of tumor angiogenesis, gene silencing, and gene-editing technology. The authors concluded that emerging novel recombinant DNA technologies and gene therapies respond to the need for new treatments in cancer. Nevertheless, further studies are needed to analyze its use in combination with other therapies in clinical practice.

Montalvo-Casimiro et al. summarized the use of epidrugs, which are novel epigenetic regulators that present new therapeutic candidates against cancer. The development of epidrugs, such as 5-azacytidine and 5-aza-2′-deoxycytidine (decitabine), Hydralazine, Vorinostat (SAHA), and Valproic acid, are proposed to enhance epigenetic therapy in cancer contributing to the development of precision medicine.

Recent advances in the application of computational molecular biology and bioinformatic approaches were discussed by Hernández-Lemus and Martinez-García. In their review, the authors emphasize the importance of the use of both high-throughput-omics data analyses and mining of extensive, well-annotated databases, which should be supplemented with experimental data and clinical validation. These interdisciplinary approaches represent a comprehensive methodology to combat some of the challenges during anticancer drug repurposing.

Cortés et al. centered their review on discussing the advantages of the knowledge of the cellular and molecular mechanisms of skin cancer, which have provided essential information for drug repurposing for this disease. The authors emphasize that the evidence from ongoing clinical trials in this regard is limited. Therefore, the authors invite researchers to expand on this topic and comment that the addition of nano-formulations could improve the efficacy of drugs to treat cancer; thus, this approach will allow repurposing known drugs to treat skin cancer.

On the other hand, Llaguno-Munive et al. provide a comprehensive review on glioma, the most common and aggressive primary tumor of the central nervous system. The authors discussed the repositioning of mifepristone, an antiprogestin, as an adjuvant drug to treat high-grade gliomas. Indeed, its effectiveness against cancer is already being analyzed in clinical trials. Also, the authors summarized previous findings that reported a synergistic action when mifepristone is combined with cisplatin or temozolomide plus radiation in cancer. Mifepristone is a repositioned drug that promises improved therapeutic efficiency and more prolonged patient survival.

Acute lymphoblastic leukemia (ALL) is known to present resistance to conventional treatment and glucocorticoids (GC). In their work, Olivas-Aguirre et al. reviewed the pharmacological strategies that reverse GC resistance. Among them, the repositioned drugs tigecycline, cannabidiol, tamoxifen, and some anthelmintics showed promising results. The authors proposed that these medications should be considered for inclusion in chemotherapeutic protocols to treat GC-resistant ALL.

Although establishing repositioned drugs appears to be a rapid strategy, several studies are undeniably required before their use in clinical practice, albeit with less time and financial resources. This topic showed that a broad range of therapeutic strategies, which span from bioinformatics analyses to cutting-edge molecular technologies, such as liposomes and CRISPR-Cas9, constitute powerful tools that are currently used in the clinic to assess the success of repositioned drugs in supplementing conventional cancer therapies.

## Author Contributions

AC-P conceived the topic. AC-P and CP-P wrote the original draft of this editorial. All authors contributed to the article and approved the submitted version.

## Conflict of Interest

The authors declare that the research was conducted in the absence of any commercial or financial relationships that could be construed as a potential conflict of interest.
